# Beyond reassurance: a validated video to support pediatric perioperative communication

**DOI:** 10.1016/j.jped.2026.101634

**Published:** 2026-10-27

**Authors:** Johan Berghmans

**Affiliations:** a Ghent University, Faculty of Medicine and Health Sciences, Department of Human Structure and Repair, Ghent, Belgium; b ZAS Cadix, Department of Anesthesiology, Antwerpen, Belgium

One of the most important and undoubtedly unsettling aspects of a child’s surgical journey is pediatric anesthesia, which in contrast happens to be the least visible as well as reassuring part. Parents are asked to entrust their beloved child to an unfamiliar team, accept temporary separation, understand fasting rules and anesthesia options, and also anticipate pain, nausea, agitation, or delayed recovery - often during a brief consultation and under considerable emotional strain. This parental anxiety is indeed not merely an accompanying inconvenience. In fact, higher parental anxiety has been associated with greater perioperative anxiety in children [1,2]. Child anxiety in turn, particularly at anesthetic induction, has been associated with increased postoperative pain and maladaptive behavioral changes [3,4]. As a result reassurance can only be attained when clear communication and accessible information are essential components of pediatric anesthesia and the foundation of Family-centered perioperative care [5].

In this issue of *Jornal de Pediatria*, Carneiro et al. describe the development and validation of a short educational video (4-minute 40-second) in Brazilian Portuguese for caregivers of children undergoing elective surgery [6]. Their work is very valuable, not because the video itself is novel, but because the content was built from the questions of caregivers, aligned with the literature, reviewed by professionals, and then assessed by members of the intended audience. Consequently their perspective moves educational material away from expert assumption and toward co-designed communication. Below, we take a more explicit look at the study’s two main strengths: the development and validation of the video.

The first lesson is simple logic: it is only common sense that families' questions should determine the starting point. Interviews with ten caregivers identified recurrent concerns about fasting, the type of anesthesia, monitoring, postoperative agitation, shivering, pain, allergy, and awareness. Yet which aspects families wish to hear more about, may vary with language and cultural background [7]. Similar information needs have been reported in other studies, suggesting that parents’ uncertainties tend to center on a consistent set of themes [8,9]. A fasting instruction, for example, must be clinically accurate, understandable to people with different levels of health literacy, and consistent with local practice. Pediatric fasting guidance has become more permissive in many settings, particularly regarding clear fluids, highlighting the need to review and update educational resources as recommendations change [10].

A second strength is the explicit validation process. Twenty-two experts rated the preliminary video, producing an average scale-level content validity index (S-CVI/Ave) of 0.94; twelve caregivers produced an S-CVI/Ave of 0.98 on a different instrument. These figures support relevance and perceived adequacy, the use of item-level indices, confidence intervals, and modified kappa add welcome transparency. Even the lowest-rated expert item about sociocultural appropriateness, prompting simplification of terminology and presentation may be more revealing than the high overall scores because it suggests that even when essential information is provided, medical terminology can make it difficult for families to understand.

Nevertheless these results require disciplined interpretation. Content validity asks whether material appears relevant and appropriate; face or semantic assessment whether it seems clear and acceptable. In reality neither method demonstrates comprehension, retention, reduced anxiety, behavioral change, fewer cancellations, or improved recovery [11]. It is clear that these very high indices stem, in part, from small convenience samples, with wide confidence intervals, and from four-point response scales without a neutral option. Caution is required as summary measures can obscure important limitations: a high S-CVI/Ave may mask weak agreement on a clinically important item, while Cronbach’s alpha describes the internal consistency of the questionnaire, not whether the educational intervention improves families’ understanding [12]. The expert and caregiver estimates came from different instruments and should not be compared as though they measured the same construct.

One methodological issue deserves particular attention. Suggestions from experts and caregivers were incorporated into the final video, but the revised version was not reassessed. This is understandable in a developmental study, yet validation properly applies to the version that participants actually evaluated. Material changes in wording, sequence, images, subtitles, or duration can improve a resource, but may also introduce new ambiguity. A brief final-cycle assessment of comprehension and usability - particularly among caregivers with limited literacy, sensory impairment, or limited familiarity with hospital care - would definitely strengthen the chain of evidence.

The distinction between validation and effectiveness is especially important because the evidence for video-based preoperative preparation is promising but heterogeneous. Systematic reviews suggest that technology-based interventions can reduce anxiety in some contexts, although interventions, timing, comparators, and outcome measures vary substantially [13]. A recent trial of the short caregiver video did not demonstrate a reduction in child anxiety at induction [14], whereas another randomized comparison found lower parental anxiety after video counseling than after brochure or verbal counseling alone [15]. These apparently divergent findings are not surprising. A video may standardize core information, but its effect depends on what it contains, when it is viewed, whether caregivers can ask questions, what their baseline anxiety is, what the impact on the child's developmental stage could be, and how the wider perioperative pathway is organized.

The most useful implementation model is therefore not ‘video versus clinician,’ but ‘video plus conversation’. A short recording can give families essential information before admission or preanesthetic consultation. Next the clinician uses the consultation to discuss the child’s medical conditions, the proposed anesthetic plan and its risks, the family’s questions, and decisions. After that the clinician can focus on individual comorbidity, the proposed anesthetic plan, material risks, caregiver concerns, and shared decisions. Understanding should be checked rather than assumed; teach-back, in which caregivers explain key instructions in their own words, constitutes one such practical approach [16]. Finally the video should invite questions and explicitly state that it supplements rather than replaces individualized assessment and consent.

It is crucial that equity remain central. Consequently it is important to note that the study excluded people whose literacy, communication, or cognitive limitations prevented participation, even though these groups may actually benefit most from carefully designed audiovisual communication [17]. Access also depends on connectivity, devices, captions, audio quality, language, and disability accommodations. Future adaptations should therefore involve users with lower literacy, different regional and cultural backgrounds, visual or hearing impairment, and varied family structures. Assessment tools such as the Patient Education Materials Assessment Tool can further complement content-validity methods by examining understandability and actionability [18]. Translation alone is certainly not sufficient: each version requires cultural adaptation, confirmation of alignment with local policy, and evaluation with its intended users.

Apart from the strengths of the research by Carneiro et al., future studies should incorporate several additional features. First, the final version should undergo usability and comprehension testing in the setting and the time at which it is intended to be used. Second, a pragmatic controlled study should evaluate outcomes that matter to families and services: knowledge, decisional confidence, caregiver and child anxiety measured with validated instruments, satisfaction, adherence to fasting instructions, day-of-surgery delays or cancellations, and unexpected calls. Clinical endpoints such as analgesic requirements, emergence delirium, and length of stay may be explored, but any trial must be adequately powered and avoid implying that communication alone determines multifactorial outcomes. Third, implementation should be evaluated [19]: who receives and views the video, its use in practice, its effect on clinician workload, whether it remains in use, and whether its benefits differ by health literacy or socio-economic background. Acceptability should also be considered prospectively as a multidimensional construct, rather than reduced to satisfaction alone [20].

As a final thought one should be aware that digital educational material benefits from continued attention after publication. As clinical guidance and local practices evolve, a designated clinical owner, regular review, and caregiver feedback can help keep the video accurate and useful. This also creates opportunities to learn how families use it and where they would still need clarification. Carneiro et al. have done more than produce a well-developed and substantiated animation: they have shown how listening to caregivers can shape clear, relevant information from the outset.

And in having gone down that path of reassurance Carneiro et al. prioritize that humanizing pediatric anesthesia means giving families honest, understandable information at the right time, recognizing the caregiver’s role in the child’s experience, and making room for individual questions. These are indeed principles central to family-centered pediatric care [5], in which clinicians work with children and caregivers to address their needs and support shared decision-making. This study offers a valuable foundation for the above-mentioned approach; hence implementing the video in routine care and assessing its effects on children and families will be important next steps.

## Data availability

N/A.

## Conflicts of interest

The author declares no conflicts of interest.
